# Transitioning to molecular diagnostics in pediatric high-grade glioma: experiences with the 2016 WHO classification of CNS tumors

**DOI:** 10.1093/noajnl/vdab113

**Published:** 2021-08-18

**Authors:** Joshua N Baugh, Gerrit H Gielen, Dannis G van Vuurden, Sophie E M Veldhuijzen van Zanten, Darren Hargrave, Maura Massimino, Veronica Biassoni, Andres Morales la Madrid, Michael Karremann, Maria Wiese, Ulrich Thomale, Geert O Janssens, André O von Bueren, Thomas Perwein, Eelco W Hoving, Torsten Pietsch, Felipe Andreiuolo, Christof M Kramm

**Affiliations:** 1 Princess Máxima Center for Pediatric Oncology, Utrecht, The Netherlands; 2 Institute of Neuropathology, Medical Center Bonn, Bonn, Germany; 3 Great Ormond Street Hospital for Children NHS Trust London, London, UK; 4 Fondazione Istituto di Ricovero e Cura a Carattere Scientifico, Istituto Nazionale dei Tumori, Milan, Italy; 5 Pediatric Neuro-Oncology, Department of Pediatric Oncology, Hospital Sant Joan de Deu, Barcelona, Spain; 6 Department of Pediatric and Adolescent Medicine, University Medical Center Mannheim, Medical Faculty Mannheim, Heidelberg University, Mannheim, Germany; 7 Division of Pediatric Hematology and Oncology, University Medical Center Goettingen, Goettingen, Germany; 8 Pediatric Neurosurgery, Charité Universitätsmedizin Berlin, Berlin, Germany; 9 Department of Radiation Oncology, University Medical Center Utrecht, Utrecht, The Netherlands; 10 Division of Pediatric Oncology and Hematology, Department of Women, Child and Adolescent, University Hospital of Geneva, Geneva, Switzerland; 11 CANSEARCH research platform in Pediatric Oncology and Hematology, Faculty of Medicine, Department of Pediatrics, Gynecology and Obstetrics, University of Geneva, Geneva, Switzerland; 12 Division of Pediatric Hematology and Oncology, Department of Pediatrics and Adolescent Medicine, Medical University of Graz, Graz, Austria; 13 Instituto Estadual Do Cérebro Paulo Niemeyer and the IDOR Institute, Rio de Janeiro, Brazil

**Keywords:** molecular diagnostics, pediatric high-grade glioma, WHO tumor classification

## Abstract

**Background:**

Pediatric neuro-oncology was profoundly changed in the wake of the 2016 revision of the WHO Classification of Tumors of the Central Nervous System. Practitioners were challenged to quickly adapt to a system of tumor classification redefined by molecular diagnostics.

**Methods:**

We designed a 22-question survey studying the impact of the revised WHO classification on pediatric high-grade glioma. The survey collected basic demographics, general attitudes, issues encountered, and opinions on pediatric subtypes. Participant answers were analyzed along socioeconomic lines utilizing the human development index (HDI) of the United Nations and membership in the group of seven (G7) world economic forum.

**Results:**

Four hundred and sixty-five participants from 53 countries were included, 187 pediatric neurooncologists (40%), 160 neuropathologists (34%), and 118 other experts (26%). When asked about pediatric high-grade glioma entities, participants from very high development countries preferred treating a patient based on genetic findings. Participants from high and medium development countries indicated using traditional histology and tumor location as mainstays for therapeutic decisions. Non-G7 countries tended to regard the introduction of molecularly characterized tumor entities as a problem for daily routine due to lack of resources.

**Conclusions:**

Our findings demonstrate an overall greater reliance and favorability to molecular diagnostics among very high development countries. A disparity in resources and access to molecular diagnostics has left some centers unable to classify pediatric high-grade glioma per the WHO classification. The forthcoming edition should strain to abate disparities in molecular diagnostic availability and work toward universal adaptation.

**Key Points**

- Very high development countries more rely on and favor molecular diagnostics.
- Centers with limited access to molecular diagnostics are unable to classify pedHGG.
- Limited molecular evaluations can make the WHO tumor classification more inclusive.

**Importance of the Study**

The revised fourth edition of WHO Classification of Tumors of the CNS drastically changed the practice of pediatric neuro-oncology. Practitioners were challenged to quickly adapt to a system of tumor classification redefined using molecular diagnostics. Our survey for the first time documents an overall greater reliance and favorability to molecular diagnostics among very high development countries. Perspectives on diagnosis and treatment of pediatric high-grade glioma differ in resource constrained settings during the molecular era.

With the revised fourth edition of the WHO Classification of Tumors of the Central Nervous System, published in 2016, the field of neuro-oncology entered the molecular era. The diagnostic approach and classification of diffuse glioma, ependymoma, and medulloblastoma, among other tumor types, underwent major changes. A rapid shift in neuropathology laboratories and neuro-oncology clinics around the world was required to implement molecular advancements and a revised tumor typing system. The intention of the revision was to increase precision and add objectivity in the identification of defined diagnostic entities that can aid the treatment of patients, and more accurately predict prognosis.^1^ The fifth edition of the WHO classification with more molecularly defined brain tumor subgroups is in the final stages of development,^2^ but key questions regarding implementation have not yet been answered. We sought to address if the implementation of molecularly defined entities into practice is adequately and equally perceived to be of added clinical benefit and supported by neurooncological professionals worldwide.

Within pediatric oncology, there is a broad disparity in financing and access to cancer care worldwide.^3^ At current levels of care, models estimate that between 2020 and 2050, 9.3 million children will die from cancer in low- and middle-income countries. This represents 84% of pediatric cancer deaths worldwide.^4^ Access to diagnostics is a well-documented problem in low- and middle-income countries.^5^ Underdiagnosis and late diagnosis being key contributors to disparities in pediatric cancer outcomes.^6^ The standard set by the WHO Classifications of Tumors of the CNS plays a pivotal role in how and if pediatric high-grade gliomas are diagnosed worldwide. Our survey on pediatric high-grade glioma (pedHGG) serves as a model disease to suggest an increasing diagnostic gap dependent on national socioeconomic environments. Knowledge on the influence of national socioeconomic environments may help increase applicability and usability of current and future pediatric CNS tumor classification.

## Methods

The survey was designed and pretested by the European Society for Paediatric Oncology High Grade Glioma Working Group (SIOPE HGG WG). An online version of the survey was built using SurveyMonkey® (San Mateo, Ca, USA). Addressees of this survey study were primarily neuropathologists, pediatric neurooncologists, neurosurgeons, radiation oncologists, neuroradiologists, and other professionals in the field of neuro-oncology. These professionals were actively approached worldwide by email on behalf of the SIOPE HGG WG between March 22 and May 8, 2019. Members of the neuro-oncology community were contacted using contacts from a prior international survey within the International Society of Neuropathology (ISN),^7^ listservs from the SIOPE Brain Tumour Group, the German Society of Pediatric Oncology and Hematology (GPOH), the German Neurooncology Working Group (NOA), the German Society of Neuropathology and Neuroanatomy (DGNN), and other international collaborators in the field of pediatric neuro-oncology. Multiple replies from the same IP and/or email address were excluded.

The survey consisted of 22 questions, 12 “Yes” or “No” questions, 8 multiple choice questions, and 2 demographic questions. Within each thematic section, we identified one key question. Respondents who failed to answer 4 out of 6 predefined key questions (including questions 1, 3, 10, 14, 16, and 17) were excluded. All key questions were dichotomous, “Yes or No.” Key questions covered subjects including (i) awareness of the revised 2016 WHO classification, (ii) awareness of the newly introduced entity diffuse midline glioma (DMG), H3K27M mutant, (iii) opinions on the upcoming 5th WHO classification regarding introducing infantile glioma, (iv) introducing pediatric subtypes for anaplastic astrocytoma and glioblastoma, (v) introducing anaplastic pilocytic astrocytoma grade III, and (vi) removing gliomatosis cerebri (Supplementary Appendix A).

The 2018 United Nations (UN) Human Development Index (HDI) was selected for the socioeconomic analysis. A country’s HDI represents the mean of three key dimensions of human development: life expectancy, education, and standard of living. The ranking system classifies countries with a HDI >.80 as very high human development, ≥0.70 to <0.80 high, <0.70 to ≥0.56 medium, and <0.56 as low human development respectively.^8^ For comparison purposes, we coupled our HDI analysis with membership in the G7 (group of the seven world leading economies).^9^ The analysis was performed using IBM SPSS Statistics version 25 (Armonk, NY, USA). Data were analyzed using Pearson’s Chi-square and Fisher’s Exact Test. Full results available in Supplementary Appendix B and C. Research involving human subjects according to the World Medical Association Declaration of Helsinki did not apply. Thus, the present study did not require an IRB review. Independent professionals, no patients, were asked for voluntary participation investigating their experience and opinion. No personal identifying data were collected and participation did not involve any advantage, disadvantage or any potential harm.

## Results

### Demographics

The questionnaire was completed by 482 participants. Seventeen respondents (4%) did not meet inclusion criteria. Participants represented a broad spectrum of specialties; 187 pediatric neurooncologists (40%), 160 neuropathologists (34%), and 118 (26%) other experts. These experts included 45 neuroradiologists (10%), 29 radiation oncologists (6%), 20 neurosurgeons (4%), 8 adult neurooncologists (2%), 7 scientists (2%), and 9 not specified (2%). Three hundred and ninety-four participants (87%) were from very high HDI countries, 44 (10%) high HDI and 15 (3%) medium HDI countries. A total of 53 countries were represented. No low development (HDI <0.56) countries participated. 261 (57%) of participants were from G7 countries (Table 1). Within the HDI groups, select countries represented a large portion (>10%) of participation. These include among the very high HDI group Germany (*n* = 115/29%) and the USA (*n* = 43/11%), within the high HDI group Brazil (*n* = 18/40%) and China (*n* = 13/24%), and within the medium HDI group India (*n* = 6/40%) and Egypt (*n* = 3/20%) (Table 2).

**Table 1.** Demographics of Participants Utilizing the United Nations Human Development Index (HDI) and Membership in the Group of Seven (G7) World Economic Forum

	Survey Participants No. (%) N = 454	Participant’s Country No. (%) N = 53	Reference List (United Nations) No. (%) N = 189
Human Development Index (HDI)			
Very High	394 (87%)	37 (70%)	59 (31%)
High	45 (10%)	9 (17%)	53 (23%)
Medium	15 (3%)	7 (13%)	39 (21%)
Low	0	0	38 (20%)
Economic Tier			
G7	261 (57%)	7 (13%)	7 (4%)
Non-G7	193 (43%)	46 (87%)	182 (96%)

**Table 2.** Representation by Country

Country	Participants
Argentina***	4 (1)
Australia***	9 (2.3)
Austria***	6 (1.5)
Belgium***	6 (1.5)
Brazil**	18 (40)
Canada***	16 (4.1)
Chile***	3 (0.8)
China**	13 (24)
Colombia**	3 (6.7)
Croatia***	2 (0.5)
Czech Rep.***	6 (1.5)
Denmark***	6 (1.5)
Egypt*	3 (20)
El Salvador*	1 (6.7)
Finland***	4 (1)
France***	20 (5.1)
Germany***	115 (29)
Greece***	4 (1)
Honduras*	1 (6.7)
Hong Kong ***	2 (0.5)
Hungary***	5 (1.3)
India*	6 (40)
Iran**	2 (4.4)
Israel***	1 (0.3)
Italy***	23 (5.8)
Japan***	20 (5.1)
Jordan**	2 (4.4)
Latvia***	1 (0.3)
Lebanon**	1 (2.2)
Lithuania***	2 (0.5)
Luxembourg***	1 (0.3)
Malta***	1 (0.3)
Mexico**	5 (11)
Morocco*	1 (6.7)
Netherlands***	10 (2.5)
New Zealand***	2 (0.5)
Norway***	7 (1.8)
Pakistan*	1 (6.7)
Poland***	2 (0.5)
Portugal***	3 (0.8)
Russia***	9 (2.3)
Slovakia***	2 (0.5)
Slovenia***	4 (1)
South Africa**	2 (13)
South Korea***	2 (0.5)
Spain***	8 (2)
Sweden***	10 (2.5)
Switzerland***	10 (2.5)
Thailand**	2 (4.4)
Turkey**	1 (2.2)
UK***	24 (6.1)
Uruguay***	1 (0.3)
USA***	43 (11)

Number of participants and % within HDI group. Medium*, High**, Very High HDI countries*** ^8^.

### Overall Experiences With the Revised Fourth Edition

**Figure 1.**
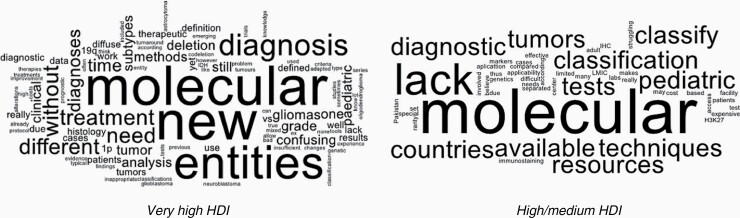
Overall experiences with the 2016 WHO Classification of CNS Tumors. Feedback from survey participants visualized using word clouds. Very High development countries (on the left) versus High/Medium development (on the right).

Participants were asked to report if the implementation of the revised WHO classification had caused problems and voluntarily provided details. Fifty-seven percent reported experiencing issues with the revision, representing 52/53 of the participating countries. 24% elaborated on their experiences in the free text portion of the survey. Feedback is visualized in Figure 1.

Very high HDI participants shared such experiences as; “some molecular tests are not readily available or validated for clinical practice,” “lack of consensus for treatment of new entities,” “new subtypes are not well known in all cooperating specialties,” “emerging new data which show new results, very often without a real influence on survival,” “a lot of the new WHO chapters do not describe pediatric gliomas well,” “treatment protocols not yet adapted to the new classification,” and “changes in criteria for diagnoses create a lot of confusion in series with retrospective evaluation of patients.”

Participants from high/medium HDI countries shared experiencing including; “because of rarity compared to adult cases, it is not cost-effective to set up tests (IHC, molecular) for pediatric tumors,” “lack of applicability due to lack of access to special techniques,” “we do not have the facility to do molecular markers and or H3K27 immunostaining,” and “resources for the classification according to the WHO 2016 are not available in many of the diagnostic labs in countries with limited resources, which makes it difficult to classify the tumors.”

### Diffuse Midline Glioma (DMG) and Diffuse Intrinsic Pontine Glioma (DIPG)

Participants from very high HDI countries more often used the diagnosis of DMG, H3K27M mutant, than high/medium HDI country participants, that is, 93% versus 65%, respectively (question 4, *P* < .001). Those from G7 countries also reported using DMG, H3K27M mutant, more often (*P* < .001). When asked about the use of DIPG at diagnosis, 59% of very high HDI respondents in comparison to 38% of high/medium HDI respondents, reported using both DIPG and DMG depending on context (question 6b, *P* = .02). G7 country participants were also more likely to use both terms (p .01). Regarding treatment of H3K27 wildtype diffuse astrocytoma, WHO grade II, of the pons that fulfills radiological criteria of DIPG, high/medium HDI respondents answered like other H3K27M high-grade gliomas (question 8c, *P* = .004). Non-G7 country participants demonstrated the same preference (*P* = .01). On the contrary, very high HDI respondents preferred personalized treatment, depending on genetic findings (53% vs 36%, respectively: question 8d, *P* = .01).

### Infants

On the introduction of infantile (high grade) glioma as a new tumor entity in the fifth WHO classification, 54% of very high HDI participants were in support, in comparison to 35% from high/medium HDI countries (question 11a, *P* = .02). Regarding classification of infantile glioma, high/medium HDI participants selected WHO grade III/IV (question 12c, *P* = .05), and very high HDI participants selected “depending on genetic findings” (question 12d, *P* = .01).

### Pediatric Subtypes

Concerning routine analysis of IDH status for (pediatric) anaplastic astrocytoma and glioblastoma, high/medium HDI participants were not in support, because of a low percentage of IDH mutant pediatric HGG (question 13a, *P* < .001). In contrast, very high HDI participants supported “obligatory” testing for all cases (question 13b, *P* < .001). G7 country participants also felt routine analysis of IDH status should be obligatory (*P* = .04). In the matter of introducing new pediatric subtypes for anaplastic astrocytoma and glioblastoma, 90% of very high HDI participants were in favor, in comparison to only 74% of the high/medium HDI group, selecting “genetic findings suggest it” (question 15b, *P* = .003).

### Gliomatosis Cerebri

Among those that support defining gliomatosis cerebri, very high HDI participants more often selected introducing a specific phenotype of an underlying glioma (question 18a, *P* = .02). While high/medium HDI participants selected introducing a specific tumor subtype (question 18b, *P* = .01, Table 3).

**Table 3.** Survey Results by Human Development Index (HDI)

		Very High HDI No. (%) N = 394	High and Medium HDI No. (%) N = 60	P Value
Q4_Do you use the diagnosis of diffuse midline glioma, H3K27M mutant?	Yes	367 (93)	39 (65)	**<.001**
	No	24 (6)	21 (35)	
	No information given	3 (1)	0	
Q6_please specify why or when you still use the term DIPG. (multiple answers possible)	Not answered	267 (68)	23 (38)	
	b. I use both terms depending on the respective context	Yes 133 (59)	Yes 14 (38)	**.02**
		No 94 (41)	No 23 (62)	
Q8_How would you treat a child (3 years and older) with a diffuse astrocytoma WHO grade II of the pons, H3K27 Wildtype, which fulfills clinical, radiological criteria of DIPG?	Not answered	23 (6)	1 (2)	
	c. Like other high-grade gliomas, H3K27M	Yes 47 (13)	Yes 16 (27)	**.004**
		No 324 (87)	No 43 (73)	
	d. Individually, depending on other genetic findings including methylation	Yes 196 (53)	Yes 21 (36)	**.01**
		No 175 (47)	No 38 (64)	
Q11_Please specify why you think there is a need to introduce a new tumor entity of “infantile glioma” for histologicallydiagnosed high-grade gliomas in infants younger than 3 years? (multiple answers possible)	Not answered	152 (39)	14 (23)	
	a. Prognosis is usually better	Yes 131 (54)	Yes 16 (35)	**.02**
		No 111 (46)	No 30 (65)	
Q12_If you think that there is indeed a need for a new tumor entity of “infantile glioma” would you classify this new entity as;	Not answered	106 (27)	10 (17)	
	c. WHO grade III/IV (depending on histological grade like it is now)	Yes 66 (23)	Yes 18 (36)	**.05**
		No 222 (77)	No 32 (64)	
	d. Individually depending on genetic findings including methylation signature	Yes 154 (54)	Yes 16 (32)	**.01**
		No 134 (46)	No 34 (68)	
Q13_What do you think about routine analysis of IDH status in pediatric anaplastic astrocytoma and glioblastoma?	Not answered	2 (.5)	0	
	a. Not adequate because of low percentage (<10%) of IDH mutant pediatric HGG	Yes 46 (12)	Yes 26 (43)	**<.001**
		No 346 (88)	No 34 (57)	
	b. Obligatory for all cases	Yes 176 (45)	Yes 12 (20)	**<.001**
		No 216 (55)	No 48 (80)	
Q15_Please specify why you think there is a need to introduce new “pediatric subtypes” for anaplastic astrocytoma andglioblastoma in children (3 years and older) and adolescents/young adults?	Not answered	120 (30)	14 (23)	
	b. Genetic findings including methylation suggest specific pediatric subtypes of anaplastic astrocytoma/ glioblastomas	Yes 246 (90)	Yes 34 (74)	**.003**
		No 28 (10)	No 12 (26)	
Q18_Please specify why you think there is still a need for diagnosis of gliomatosis cerebri with typical neuroradiological features of diffuse growth pattern involving two and more cerebral lobes?”	Not answered	159 (40)	18 (30)	
	a. Diagnosis in the renaming of a specific phenotype of an underlying glioma.	Yes 134 (57)	Yes 16 (38)	**.02**
		No 101 (43)	No 26 (62)	
	b. Diagnosis in the renaming of a specific tumor subtype of its own for an underlying glioma histology	Yes 44 (19)	Yes 16 (38)	**.01**
		No 191 (81)	No 26 (62)	

Only significant results displayed. Full length survey available in Supplementary Appendix A and results in Supplementary Appendix B.

## Discussion

Perceptions among the neuro-oncology community of the 2016 WHO Classification for CNS Tumors are not well documented, and the new fifth edition will soon be published. Our study provides input from nearly 500 neurooncological experts, representing 53 countries and 8 disciplines. Results of this survey are the first to document international differences in acceptance and implementation along socioeconomic lines of the 2016 revised fourth edition, where molecular diagnostics were introduced for the first time as basis for new tumor entities and subentities. The process of adoption and adaptation has not been the same in countries with a highly developed national health system, as it has been in countries with much fewer financial and medical resources.

Our findings demonstrated an overall greater reliance and favorability among very high HDI country participants to genetic testing. Participants in our study from very high HDI countries were significantly more likely to treat a patient individually based on genetic findings. This applied to how they would treat an infantile glioma and H3K27 wildtype DIPG, for example, whereas high and medium development countries chose using conventional grading systems based on histology and tumor location. Furthermore, when asked about the use of routine IDH1 analysis for pediatric anaplastic astrocytoma and glioblastoma, only very high HDI countries considered this obligatory. The same divide was evident in the use of molecular diagnosis of diffuse midline glioma, H3K27M mutant. Significantly more very high HDI country participants reported using the DMG diagnosis and differentiating between DMG and the radiological diagnosis of DIPG depending on the context.

Socioeconomic differences and resulting attitudes as suggested in our study were largely a distillation of whether participants have access to molecular diagnostic tools. Our survey documents participants from lesser developed and some high and even very high development countries find access to molecular test to be a barrier. A 2016 survey by the International Society of Neuropathology (ISN) underlined the issues surrounding access to molecular diagnostics. They found 25% of participating countries and 79/314 neuropathology centers declared not to have access to molecular diagnostics for brain tumors. Furthermore, 12% of the neuropathologists surveyed claimed to be unfamiliar with molecular techniques.^7^ Disparities in diagnostic usage stem from a lack of availability, accessibility, or acceptability.^10^ In the context of molecular diagnostics for CNS tumors, evident in our survey is that they are in fact available and accepted, however not internationally accessible.

The ambition of the World Health Organization, with 194 Member States, is “to achieve better health for everyone, everywhere, united in a shared commitment”.^11^ How WHO sponsored working groups, such as cIMPACT-NOW (Consortium to Inform Molecular and Practical Approaches to CNS Tumor Taxonomy), which try to adapt and explain identified issues with the current 2016 WHO classification,^12^ can recommend solutions to abate issues of access to molecular tests remains to be seen. It has been acknowledged there would be a transition period during the adaption of a molecular based system,^1^ however our survey results provide a glimpse into the current state of affairs. The development of an “integrated system” approach that uses both phenotype and genotype for CNS tumors is meant in part as a stop gap during the transition to a more genotype-based system, yet some diagnoses already required genotyping.^2^ Our survey results demonstrate the mechanisms to introduce a genetic layer of neuropathological diagnoses have not been sufficient so far to bridge the resource gap in a large part of the world. As a result, many centers in lower income settings cannot adequately diagnose pediatric high-grade glioma patients *per* the WHO 2016 criteria.

To make the WHO classification of CNS tumors more inclusive, alternative recommendations can be made for limited molecular evaluations by widely accessible tests such as FISH analysis or immunohistochemical surrogates, correlated with histology and complementary imaging. Guidelines to limit molecular testing in the setting of resource constraints and limited access to diagnostics are needed and would also be helpful for more general tumor types lacking effective targeted therapies. Should the WHO classification always consider if there is a clinical impact for each genotype? If not, how can phenotypic tumor typing still be useful and integrated moving forward? Participants in our survey mention a clinical disconnect between the WHO diagnostic requirements for genotyping and implications for therapy. Why is that the case and how can it be remedied?

A WHO CNS tumor classification that predominantly incorporates clinically significant phenotypes would enable centers without access to advanced molecular diagnostics to participate in the global neuro-oncology community more actively. Expanding cancer networks and population-based cancer registries to include more low-and middle-income countries, will increase access to diagnostic services, treatments, and foster research.^4^ In pediatric HGG, rare disease registries that also function as networks, such as the SIOPE DIPG/DMG Registry and the International DIPG/DMG Registry, provide promising avenues to increase inclusion of countries beyond very highly developed countries. These organizations provide an infrastructure and international network of neuro-oncology expertise with the goal of enabling interdisciplinary and translational projects specifically for DIPG/DMG.^13,14^ In collaboration with organizations like the WHO, pediatric cancer registries/networks can aid the rapid deployment of neuropathological expertise, molecular diagnostics, and treatments for high-grade gliomas into high, middle and low-income countries. Bold research, financing, and implementation agendas are needed to bridge disparities in pediatric neuro-oncology cancer care and control worldwide.^3^ Suggestive from our survey, the WHO Classification of CNS Tumors can play a role in perpetuating or eliminating disparities within the neuro-oncology community.

Although our study included voices from several underrepresented and less developed countries it should be acknowledged that participation from these countries remains a limitation. We had 13% representation from high/medium development countries, comprising 16 countries, and no representatives from low-income countries. Furthermore, sometimes only one or two participants answered for each high/medium HDI countries, thus, subgroup analyses of for example views between clinical neurooncologists or neuropathologists could not be made. Nevertheless, our limited socioeconomic and geographic diversity is reflective of the disparities within pediatric cancer care worldwide, as outlined above.^3^ Despite these limitations, our study raises the most geographically and socioeconomically diverse set of voices to date from the pediatric neuro-oncology community.

## Conclusions

The 2016 revision of WHO classification drastically changed the practice of pediatric neuro-oncology. Around the world, practitioners were challenged to quickly adapt to a system of tumor classification redefined using molecular diagnostics. Our survey for the first time documents how disparities in access to molecular diagnostics can shape the implementation of the WHO 2016 tumor classification, and how perspectives toward diagnosis and treatment can differ in resource constrained settings during the molecular era. The forthcoming fifth edition should strain to abate disparities in molecular diagnosis between rich and poor countries and define a “minimum needed” molecular panel for each histotype.

## Supplementary Material

vdab113_suppl_Supplementary_Materials

## Notes

**Authorship Statement**. Conception and design: J.N.B., G.H.G., F.A., C.M.K. Acquisition of data: J.N.B., G.H.G., C.M.K. Analysis and interpretation of data: J.N.B., G.H.G., C.M.K., D.G.V., S.E.M.V.Z. Drafting or revising critically for important intellectual content: All authors. Final approval of the version to be published: All authors.
