# Supplementary material for: Transitioning to molecular diagnostics in pediatric high-grade glioma: experiences with the 2016 WHO classification of CNS tumors
Source: Neurooncol Adv. 2021 Aug 18;3(1):vdab113. doi: 10.1093/noajnl/vdab113 (PMC8478775; doi:10.1093/noajnl/vdab113)
Supplement: vdab113_suppl_Supplementary_Materials [file vdab113_suppl_supplementary_materials.docx]

**Appendix A.** Full Length Survey Copy

**Pediatric HGG and YOUR experience with the revised WHO classification**

1. Are you aware of the revision of the WHO Classification of Tumours of the Central Nervous System that

occurred in 2016?

*If you are a neuropathologist who needs to work with the revised classification please don't feel offended and*

*continue :) ...*

- No
- Yes

2. Do you use the revised WHO Classification in your daily practice?

- No
- Yes

3. Are you aware of the newly introduced tumour entity “diffuse midline glioma, H3K27M mutant (WHO grade

IV)”?

- No
- Yes

4. Do you use the diagnosis of diffuse midline glioma, H3K27M mutant?

- No
- Yes

5. Do you still prefer DIPG (“diffuse intrinsic pontine glioma”) as neuroradiological/clinical diagnosis instead of

diffuse midline glioma, H3K27M mutant, when located within the pons?

- No
- Yes

6. **If you answered YES to the previous question** (*"Do you still prefer DIPG as neuroradiological/clinical*

*diagnosis instead of diffuse midline glioma ...?"*), please specify why or when you still use the term DIPG

(several answers are possible):

- DIPG is a well defined and established diagnosis/diagnostic term
- I use both terms depending on the respective context
- Patients can better understand DIPG as diagnosis than diffuse midline glioma, H3K27M mutant
- Diffuse midline glioma, H3K27M mutant, does not cover all DIPG
- Any other answer?_____________

7. Do you believe there is an entity of DIPG, **H3K27 WILDTYPE**, WHO IV?

- No
- Yes

8. How would you treat a child (3 years and older) with a diffuse astrocytoma WHO grade II of the pons,

**H3K27 WILDTYPE**, which fulfils clinical/neuroradiological criteria of DIPG?

- Like a low grade glioma
- Like a diffuse midline glioma, H3K27M mutant
- If using different protocols for diffuse midline gliomas, H3K27M mutant, and other high grade gliomas: Like other high grade
- gliomas
- Individually, depending on other genetic findings including methylation

9. Do you think there is a need to introduce a new tumour entity of **“Diffuse midline glioma of the pons,**

**H3K27 WILDTYPE (WHO grade IV)**” with typical neuroradiological features of a DIPG?

- No
- Yes

10. Do you think there is a need to introduce a new tumour entity of “infantile glioma” for histologically

diagnosed high grade gliomas in infants younger than 3 years?

No

Yes

11. **If you answered YES to the previous question** (*"Do you think there is a need to introduce a new tumour*

*entity of “infantile glioma” ..."*), please specify why (several answers are possible):

- Prognosis is usually significantly better
- Genetic findings including methylation suggest a tumour entity of its own
- Therapy is usually different from high grade gliomas of older children and adults
- Any other reason? _____________

12. If you think that there is indeed a need for a new tumour entity of “infantile glioma” would you classify this

new entity as

- WHO grade I
- WHO grade II
- WHO grade III/IV (depending on histological grade like it is now)
- Individually depending on genetic findings including methylation signature
- Without a defined WHO grade

13. What do you think about routine analysis of IDH status in paediatric anaplastic astrocytomas and

glioblastomas?

- Not adequate because of low percentage (<10%) of IDH mutant paediatric HGG
- Obligatory for all cases
- Only if sufficient tumor material is available
- I don´t know
- Any other comment? _____________

14. Do you think there is a need to introduce new “paediatric subtypes” for anaplastic astrocytomas and

glioblastomas in children (3 years and older) and adolescents/young adults?

- No
- Yes

15. **If you answered YES to the previous question** (*"Do you think there is a need to introduce new*

*“paediatric subtypes” for anaplastic astrocytomas and glioblastomas ..."*), please specify why (several answers

are possible):

- Prognosis is usually better than in older adults
- Genetic findings including methylation suggest specific paediatric subtypes of anaplastic astrocytomas/glioblastomas
- Any other reason? _____________

16. Do you think there is a need to introduce a new tumour entity of “Anaplastic pilocytic astrocytoma (WHO

grade III)” or “Anaplastic astrocytoma with piloid features (WHO grade III)”, respectively, for pilocytic

astrocytomas with anaplastic features?

- No
- Yes

17. Do you think there is still a need for diagnosis of gliomatosis cerebri with typical neuroradiologcal features

of diffuse growth pattern involving two and more cerebral lobes ?

- No
- Yes

18. **If you answered YES to the previous question** (*"Do you think there is still a need for diagnosis of*

*gliomatosis cerebri ..."*), please specify (several answers are possible):

- Diagnosis in the meaning of a SPECIFIC PHENOTYPE of an underlying glioma, but not as a tumour subtype or entity of its own
- Diagnosis in the meaning of a SPECIFIC TUMOUR SUBTYPE of its own for an underlying glioma histology
- Diagnosis in the meaning of a TUMOUR ENTITY of its own independently of an underlying glioma histology
- Any other suggestions? _____________

19. In summary, has the implementation of the revised WHO Classification caused any problems?

- No
- Yes

20. **If you answered YES to the previous question** (*"In summary, has the implementation of the revised*

*WHO Classification caused any problems?"*), please specify your relevant issues (several answers are

possible):

- Introduction of new tumour entities
- Abolishment of tumour entities
- Renaming of tumour entities
- Insufficient diagnostic definitions of tumour entities
- Diagnostic definitions are less relevant for pediatric than for adult neurooncology
- Diagnostic definitions are sometimes hard to explain to patients/parents
- Any other problems? _____________

21. What is your field of expertise?

- Paediatric Oncologist/Paediatric Neurooncologist
- Neuropathologist
- Neurosurgeon
- Radiotherapist
- Radiologist/Neuroradiologist
- Scientist/Biologist/Physician Scientist
- Any other field of expertise? _____________

22. In which country are you working?

and________________________________ YOUR experience

**Appendix B.** Full Survey Results by HDI. Participant No. (% within HDI)

|  | | Very High HDI  No. (%)  N=394 | High and Medium HDI No. (%)  N=60 | P value |
| --- | --- | --- | --- | --- |
| Q1_Are you aware of the revision of the WHO Classification of Tumors of the Central Nervous System that occurred in 2016? | Yes | 375 (95) | 58 (97) | 1.0 |
|  | No | 19 (5) | 2(3) |  |
| Q2_Do you use the revised WHO Classification in your daily practice? | Yes | 366 (93) | 55 (92) | .70 |
|  | No | 24 (6) | 4 (7) |  |
|  | No information given | 4 (1) | 1 (2) |  |
| Q3_Are you aware of the newly introduced tumour entity “diffuse midline glioma, H3K27M mutant (WHO grade IV)”? | Yes | 380 (96) | 56 (93) | .43 |
|  | No | 12 (3) | 4 (7) |  |
|  | No information given | 2 (1) | 0 |  |
| Q4_Do you use the diagnosis of diffuse midline glioma, H3K27M mutant? | Yes | 367 (93) | 39 (65) | **<.001** |
|  | No | 24 (6) | 21 (35) |  |
|  | No information given | 3 (1) | 0 |  |
| Q5_Do you still prefer DIPG as radiological/clinical diagnosis instead of  diffuse midline glioma, H3K27M mutant, when located within the pons? | Yes | 180 (46) | 32 (53) | .50 |
|  | No | 212 (54) | 28 (47) |  |
|  | No information given | 2 (.5) | 0 |  |
| Q6_If you answered YES to the previous question, please specify why or when you still use the term DIPG.  (multiple answers possible) | Not answered (% total HDI) | 267 (68) | 23 (38) |  |
|  | a. DIPG is a well defined and established diagnosis/diagnostic term | Yes 104 (46)  No 123 (54) | Yes 14 (38)  No 23 (62) | .40 |
|  | b. I use both terms depending on the respective context | Yes 133 (59)  No 94 (41) | Yes 14 (38)  No 23 (62) | **.02** |
|  | c. Patients can better understand DIPG as diagnosis than diffuse midline glioma, H3K27M mutant | Yes 74 (33)  No 153 (67) | Yes 7 (19)  No 30 (81) | .09 |
|  | d. Diffuse midline glioma, H3K27M mutant, does not cover all DIPG | Yes 76 (34)  No 151 (66) | Yes 13 (35)  No 24 (65) | .84 |
| Q7_Do you believe there is an entity of DIPG, H3K27 WILDTYPE, WHO IV? | Yes | 281 (71) | 50 (83) | .15 |
|  | No | 91 (23) | 9 (15) |  |
|  | No information given | 22 (6) | 1 (2) |  |
| Q8_How would you treat a child (3 years and older) with a diffuse astrocytoma WHO grade II of the pons,  H3K27 WILDTYPE, which fulfills clinical/radiological criteria of DIPG? | Not answered (% total HDI) | 23 (6) | 1 (2) |  |
|  | a. Like a low grade glioma | Yes 36 (10)  No 335 (90) | Yes 6 (10)  No (90) | .91 |
|  | b. Like a diffuse midline glioma, H3k27M mutant | Yes 92 (25)  No 279 (75) | Yes 16 (27)  No 43 (73) | .70 |
|  | c. Like other high grade gliomas, H3K27M | Yes 47 (13)  No 324 (87) | Yes 16 (27)  No 43 (73) | **.004** |
|  | d. Individually, depending on other genetic findings including methylation | Yes 196 (53)  No 175 (47) | Yes 21 (36)  No 38 (64) | **.01** |
| Q9_Do you think there is a need to introduce a new tumour entity of “Diffuse midline glioma of the pons,  H3K27 WILDTYPE (WHO grade IV)” with typical neuroradiological features of a DIPG? | Yes | 221 (56) | 42 (70) | .11 |
|  | No | 152 (39) | 17 (28) |  |
|  | No information given | 21 (5) | 1 (2) |  |
| Q10_Do you think there is a need to introduce a new tumour entity of “infantile glioma” for histologically  diagnosed high grade gliomas in infants younger than 3 years? | Yes | 237 (60) | 44 (73) | .11 |
|  | No | 147 (37) | 16 (27) |  |
|  | No information given | 10 (3) | 0 |  |
| Q11_If you answered YES to the previous question, please specify why (multiple answers possible) | Not answered (% total HDI) | 152 (39) | 14 (23) |  |
|  | a. Prognosis is usually better | Yes 131 (54)  No 111 (46) | Yes 16 (35)  No 30 (65) | **.02** |
|  | b. Genetic findings including methylation suggest a tumour entity of its own | Yes 151 (62)  No 91 (38) | Yes 30 (65)  No 16 (35) | .72 |
|  | c. Therapy is usually from high grade gliomas of older children and adults | Yes 132 (55)  No 110 (45) | Yes 19 (41)  No 27 (59) | .10 |
| Q12_If you think that there  is indeed a need for a new tumour entity of “infantile glioma” would you classify this new entity as; | Not answered (% total HDI) | 106 (27) | 10 (17) |  |
|  | a. WHO grade I | Yes 1 (.3)  No 287 (99) | Yes 0 (0)  No 50 (100) | 1.0 |
|  | b. WHO grade II | Yes 10 (4)  No 278 (96) | Yes 3 (6)  No 47 (94) | .42 |
|  | c. WHO grade III/IV (depending on histological grade like it is now) | Yes 66 (23)  No 222 (77) | Yes 18 (36)  No 32 (64) | **.05** |
|  | d. Individually depending on genetic findings including methylation signature | Yes 154 (54)  No 134 (46) | Yes 16 (32)  No 34 (68) | **.01** |
|  | e. Without a defined WHO grade | Yes 57 (20)  No 231 (80) | Yes 13 (26)  No 37 (74) | .32 |
| Q13_What do you think about routine analysis of IDH status in paediatric anaplastic astrocytoma  and glioblastoma? | Not answered (% total HDI) | 2 (.5) | 0 |  |
|  | a. Not adequate because of low percentage (<10%) of IDH mutant paediatric HGG | Yes 46 (12)  No 346 (88) | Yes 26 (43)  No 34 (57) | **<.001** |
|  | b. Obligatory for all cases | Yes 176 (45)  No 216 (55) | Yes 12 (20)  No 48 (80) | **<.001** |
|  | c. Only if sufficient tumour material is available | Yes 81 (21)  No 311 (79) | Yes 12 (20)  No 48 (80) | .91 |
|  | d. I don´t know | Yes 44 (11)  No 348 (89) | Yes 8 (13)  No 52 (87) | .63 |
|  |  |  |  |  |
| Q14_Do you think there is a need to introduce new “paediatric subtypes” for anaplastic astrocytoma and  glioblastoma in children (3 years and older) and adolescents/young adults? | Yes | 268 (68) | 44 (73) | .63 |
|  | No | 117 (30) | 16 (27) |  |
|  | No information given | 9 (2) | 0 |  |
| Q15_If you answered YES to the previous question, please specify why (multiple answers possible) | Not answered (% total HDI) | 120 (30) | 14 (23) |  |
|  | a. Prognosis is usually better than in adults. | Yes 82 (30)  No 192 (70) | Yes 20 (44)  No 26 (56) | .07 |
|  | b. Genetic findings including methylation suggest specific paediatric subtypes of anaplastic astrocytoma/ glioblastomas | Yes 246 (90)  No 28 (10) | Yes 34 (74)  No 12 (26) | **.003** |
| Q16_Do you think there is a need to introduce a new tumour entity of “Anaplastic pilocytic astrocytoma (WHO  grade III)” or “Anaplastic astrocytoma with piloid features (WHO grade III)”, respectively, for pilocytic  astrocytoma with anaplastic features? | Yes | 245 (62) | 46 (77) | .08 |
|  | No | 133 (34) | 12 (20) |  |
|  | No information given | 16 (4) | 2 (3) |  |
| Q17_Do you think there is still a need for diagnosis of gliomatosis cerebri with typical neuroradiological features of diffuse growth pattern involving two and more cerebral lobes? | Yes | 224 (57) | 42 (70) | .17 |
|  | No | 165 (42) | 18 (30) |  |
|  | No information given | 5 (1) | 0 |  |
| Q18_If you answered YES to the previous question, please specify (multiple answers possible) | Not answered (% total HDI) | 159 (40) | 18 (30) |  |
|  | a. Diagnosis in the renaming of a SPECFIC PHENOTYPE of an underlying glioma. | Yes 134 (57)  No 101 (43) | Yes 16 (38)  No 26 (62) | **.02** |
|  | b. Diagnosis in the renaming of a SPECIFIC TUMOUR SUBTYPE of its own for an underlying glioma histology | Yes 44 (19)  No 191 (81) | Yes 16 (38)  No 26 (62) | **.01** |
|  | c. Diagnosis in the renaming of a TUMOUR ENTITY of its own independently of an underlying glioma | Yes 46 (20)  No 189 (80) | Yes 8 (19)  No 34 (81) | .94 |
| Q19_In summary, has the implementation of the revised WHO Classification caused any problems? | Yes | 220 (56) | 43 (72) | .07 |
|  | No | 169 (43) | 17 (28) |  |
|  | No information given | 5 (1) | 0 |  |
| Q20_If you answered YES to the previous question, please specify your relevant issues (multiple answers possible) |  |  |  |  |
|  | Not answered (% total HDI) | 156 (40) | 15 (25) |  |
|  | a. Introduction of new tumour entities. | Yes 82 (35)  No 156 (65) | Yes 15 (33)  No 30 (67) | .89 |
|  | b. Abolishment of tumour entities | Yes 70 (29)  No 168 (71) | Yes 11 (24)  No 34 (76) | .50 |
|  | c. Renaming of tumour entities | Yes 77 (32)  No 161 (68) | Yes 16 (36)  No 29 (64) | .68 |
|  | d. Insufficient diagnostic definitions of tumour entities. | Yes 108 (45)  No 130 (55) | Yes 16 (36)  No 29 (64) | .22 |
|  | e. Diagnostic definitions are less relevant for paediatric than for adult neurooncology. | Yes 92 (39)  No 146 (61) | Yes 16 (36)  No 29 (64) | .70 |
|  | f. Diagnostic definitions are sometimes hard to explain to patients/parents | Yes 72 (30)  No 166 (70) | Yes 17 (38)  No 28 (62) | .32 |

**Appendix C.** Full Survey Results G7 vs Non-G7. Participant No. (% within HDI)

|  | | G7 Count (%)  N=261 | Non-G7 Count (%)  N=193 | P value |
| --- | --- | --- | --- | --- |
| Q1_Are you aware of the revision of the WHO Classification of Tumors of the Central Nervous System that occurred in 2016? | Yes | 246 (94) | 187 (97) | .19 |
|  | No | 15 (6) | 6 (3) |  |
| Q2_Do you use the revised WHO Classification in your daily practice? | Yes | 240 (92) | 181 (94) | .38 |
|  | No | 19 (7) | 9 (5) |  |
|  | No information given | 2 (1) | 3 (1) |  |
| Q3_Are you aware of the newly introduced tumour entity “diffuse midline glioma, H3K27M mutant (WHO grade IV)”? | Yes | 253 (97) | 183 (95) | .11 |
|  | No | 6 (2) | 10 (5) |  |
|  | No information given | 2 (1) | 0 |  |
| Q4_Do you use the diagnosis of diffuse midline glioma, H3K27M mutant? | Yes | 246 (94) | 160 (83) | **<.001** |
|  | No | 13 (5) | 32 (17) |  |
|  | No information given | 2 | 1 |  |
| Q5_Do you still prefer DIPG as radiological/clinical diagnosis instead of  diffuse midline glioma, H3K27M mutant, when located within the pons? | Yes | 111 (42) | 101 (52) | **.05** |
|  | No | 148 (57) | 92 (48) |  |
|  | No information given | 2 (1) | 0 |  |
| Q6_If you answered YES to the previous question, please specify why or when you still use the term DIPG.  (multiple answers possible) | Not answered (% total HDI) | 120 (46) | 70 (36) |  |
|  | a.DIPG is a well defined and established diagnosis/diagnostic term | Yes 62 (44)  No 79 (56) | Yes 56 (45)  No 67 (55) | .80 |
|  | b.I use both terms depending on the respective context | Yes 89 (63)  No 52 (37) | Yes 58 (47)  No 65 (53) | **.01** |
|  | c.Patients can better understand DIPG as diagnosis than diffuse midline glioma, H3K27M mutant | Yes 50 (35)  No 91 (65) | Yes 31 (25)  No 92 (75) | .07 |
|  | d. Diffuse midline glioma, H3K27M mutant, does not cover all DIPG | Yes 45 (32)  No 96 (68) | Yes 44 (36)  No 79 (64) | .51 |
| Q7_Do you believe there is an entity of DIPG, H3K27 WILDTYPE, WHO IV? | Yes | 180 (69) | 151 (78) | **.03** |
|  | No | 69 (26) | 31 (16) |  |
|  | No information given | 12 (5) | 11 (6) |  |
| Q8_How would you treat a child (3 years and older) with a diffuse astrocytoma WHO grade II of the pons,  H3K27 WILDTYPE, which fulfills clinical/radiological criteria of DIPG? | Not answered (% total HDI) | 16 (6) | 13 (7) |  |
|  | a. Like a low grade glioma | Yes 23 (9)  No 227 (91) | Yes 19 (11)  No 161 (89) | .64 |
|  | b. Like a diffuse midline glioma, H3k27M mutant | Yes 70 (28)  No 180 (72) | Yes 38 (21)  No 142 (79) | .10 |
|  | c. Like other high grade gliomas, H3K27M | Yes 27 (11)  No 223 (89) | Yes 36 (20)  No 144 (80) | **.01** |
|  | d. Individually, depending on other genetic findings including methylation | Yes 130 (52)  No 120 (48) | Yes 87 (48)  No 93 (52) | .45 |
| Q9_Do you think there is a need to introduce a new tumour entity of “Diffuse midline glioma of the pons,  H3K27 WILDTYPE (WHO grade IV)” with typical neuroradiological features of a DIPG? | Yes | 144 (55) | 119 (62) | .20 |
|  | No | 106 (41) | 63 (33) |  |
|  | No information given | 11 (4) | 11 (5) |  |
| Q10_Do you think there is a need to introduce a new tumour entity of “infantile glioma” for histologically  diagnosed high grade gliomas in infants younger than 3 years? | Yes | 143 (55) | 138 (72) | **.001** |
|  | No | 111 (42) | 52 (27) |  |
|  | No information given | 7 (3) | 3 (2) |  |
| Q11_If you answered YES to the previous question, please specify why (multiple answers possible) | Not answered (% total HDI) | 114 (44) | 52 (27) |  |
|  | a. Prognosis is usually better | Yes 79 (54)  No 68 (46) | Yes 68 (48)  No 73 (52) | .35 |
|  | b. Genetic findings including methylation suggest a tumour entity of its own | Yes 97 (66)  No 50 (34) | Yes 84 (60)  No 57 (40) | .26 |
|  | c. Therapy is usually from high grade gliomas of older children and adults | Yes 81 (55)  No 66 (45) | Yes 70 (50)  No 71 (50) | .35 |
| Q12_If you think that there  is indeed a need for a new tumour entity of “infantile glioma” would you classify this new entity as; | Not answered (% total HDI) | 78 (30) | 38 (20) |  |
|  | a. WHO grade I | Yes 1 (.5)  No 182 (99) | Yes 0  No 155(100) | .36 |
|  | b. WHO grade II | Yes 6 (3)  No 177 (97) | Yes 7 (5)  No 148 (95) | .56 |
|  | c. WHO grade III/IV (depending on histological grade like it is now) | Yes 50 (27)  No 133 (73) | Yes 34 (22)  No 121 (78) | .25 |
|  | d. Individually depending on genetic findings including methylation signature | Yes 94 (51)  No 89 (49) | Yes 76 (49)  No 79 (51) | .67 |
|  | e. Without a defined WHO grade | Yes 32 (17)  No 151 (83) | Yes 38 (24)  No 117 (76) | .11 |
| Q13_What do you think about routine analysis of IDH status in paediatric anaplastic astrocytoma  and glioblastoma? | Not answered (% total HDI) | 1 (.4) | 1 (5) |  |
|  | a. Not adequate because of low percentage (<10%) of IDH mutant paediatric HGG | Yes 27 (10)  No 233 (90) | Yes 45 (23)  No 147 (77) | **<.001** |
|  | b. Obligatory for all cases | Yes 119 (46)  No 141 (54) | Yes 69 (36)  No 123 (64) | **.04** |
|  | c. Only if sufficient tumour material is available | Yes 54 (21)  No 206 (79) | Yes 39 (20)  No 153 (80) | .91 |
|  | d. I don´t know | Yes 28 (11)  No 232 (89) | Yes 24 (13)  No 168 (87) | .60 |
|  |  |  |  |  |
| Q14_Do you think there is a need to introduce new “paediatric subtypes” for anaplastic astrocytoma and  glioblastoma in children (3 years and older) and adolescents/young adults? | Yes | 174 (67) | 138 (71) | .30 |
|  | No | 83 (32) | 50 (26) |  |
|  | No information given | 4 (2) | 5 (3) |  |
| Q15_If you answered YES to the previous question, please specify why (multiple answers possible) | Not answered (% total HDI) | 83 (32) | 51 (26) |  |
|  | a. Prognosis is usually better than in adults. | Yes 58 (33)  No 120 (67) | Yes 44 (31)  No 98 (69) | .76 |
|  | b. Genetic findings including methylation suggest specific paediatric subtypes of anaplastic astrocytoma/ glioblastomas | Yes 158 (89)  No 20 (11) | Yes 122(86)  No 20 (14) | .44 |
| Q16_Do you think there is a need to introduce a new tumour entity of “Anaplastic pilocytic astrocytoma (WHO  grade III)” or “Anaplastic astrocytoma with piloid features (WHO grade III)”, respectively, for pilocytic  astrocytoma with anaplastic features? | Yes | 165 (63) | 126 (65) | .65 |
|  | No | 87 (33) | 58 (30) |  |
|  | No information given | 9 (4) | 9 (5) |  |
| Q17_Do you think there is still a need for diagnosis of gliomatosis cerebri with typical neuroradiological features of diffuse growth pattern involving two and more cerebral lobes? | Yes | 140 (54) | 126 (65) | **.004** |
|  | No | 120 (46) | 63 (33) |  |
|  | No information given | 1 | 4 (2) |  |
| Q18A_If you answered YES to the previous question, please specify (multiple answers possible) | Not answered (% total HDI) | 112 (43) | 65 (34) |  |
|  | a. Diagnosis in the renaming of a SPECFIC PHENOTYPE of an underlying glioma. | Yes 85 (57)  No 64 (43) | Yes 65 (51)  No 63 (49) | .30 |
|  | b. Diagnosis in the renaming of a SPECIFIC TUMOUR SUBTYPE of its own for an underlying glioma histology | Yes 29 (19)  No 120 (81) | Yes 31 (24)  No 97 (76) | .34 |
|  | c. Diagnosis in the renaming of a TUMOUR ENTITY of its own independently of an underlying glioma | Yes 25 (17)  No 124 (83) | Yes 29 (23)  No 99 (77) | .22 |
| Q19_In summary, has the implementation of the revised WHO Classification caused any problems? | Yes | 146 (56) | 117 (61) | .58 |
|  | No | 112 (43) | 74 (38) |  |
|  | No information given | 3 (1) | 2 (1) |  |
| Q20_If you answered YES to the previous question, please specify your relevant issues (multiple answers possible) |  |  |  |  |
|  | Not answered (% total HDI) | 105 (40) | 66 (34) |  |
|  | a. Introduction of new tumour entities. | Yes 45 (29)  No 111 (71) | Yes 52 (41)  No 75 (59) | **.03** |
|  | b. Abolishment of tumour entities | Yes 49 (31)  No 107 (69) | Yes 32 (25)  No 95 (75) | .25 |
|  | c. Renaming of tumour entities | Yes 47 (30)  No 109 (70) | Yes 46 (36)  No 81 (64) | .28 |
|  | d. Insufficient diagnostic definitions of tumour entities. | Yes 71 (45)  No 85 (55) | Yes 53 (42)  No 74 (58) | .52 |
|  | e. Diagnostic definitions are less relevant for paediatric than for adult neurooncology. | Yes 56 (36)  No 100 (64) | Yes 52 (41)  No 75 (59) | .40 |
|  | f. diagnostic definitions are sometimes hard to explain to patients/parents | Yes 54 (35)  No 102 (65) | Yes 35 (28)  No 92 (72) | .20 |
